# Prediction of human embryo ploidy based on morphokinetic parameters: Investigating the use of time-lapse imaging for non-invasive embryo selection

**DOI:** 10.5935/1518-0557.20250035

**Published:** 2025

**Authors:** Bruno Ramalho de Carvalho, Letícia Saldanha Camargos Aires, Maria Fernanda Araújo Lima, Mariana Valadares Bittar, Íris de Oliveira Cabral, Luiz Carlos Pinheiro Junior, Adelino Amaral Silva

**Affiliations:** 1 Bruno Ramalho Reprodução Humana, Brasília, DF, Brazil; 2 Centro Universitário de Brasília - CEUB, Brasília, DF, Brazil; 3 Genesis Centro de Assistência em Reprodução Humana, Brasília, DF, Brazil

**Keywords:** IVF, embryo morphokinetics, aneuploidy, embryo quality, time-lapse imaging

## Abstract

**Objective:**

To evaluate the relationship between time-lapse (TL) imaging parameters and the ploidy of human blastocysts.

**Methods:**

This observational study retrospectively analyzes time-lapse images from 84 blastocysts tested for aneuploidies, focusing on morphokinetic evaluation based on cell division (st2, t2, t3, t4, t5, t8, tSC) and blastocyst formation parameters (tSB, tB).

**Results:**

Among the TL parameters, t5, t2-st2, cc3 (t5-t3), t5-t2, s3 (t8-t5), and tB-tSB showed a significant association with ploidy, being shorter for euploid blastocysts. The respective median times/intervals for euploid and aneuploid embryos were as follows: t5, 46.5 *vs*. 49.6 (*p*=0.015); t2-st2, 2.5 *vs*. 2.25 (*p*=0.0236); cc3, 11.7 *vs*. 13.3 (*p*=0.046); t5-t2, 22.1 *vs*. 24.4 (*p*=0.0185); s3, 10.9 *vs*. 3.3 (*p*=0.019); and tB-tSB, 7.6 *vs*. 9.9 (*p*=0.0084). ROC curves identified a moderate predictive ability of the parameters to discern euploid embryos. Additionally, initial cytoplasmic movements prior to the first cell cleavage were noted; pattern 1 (vibration-like movements), pattern 2 (circular wave movements), or pattern 0 (the absence of detectable movements) were not significantly correlated with ploidy.

**Conclusions:**

Euploid blastocysts reach certain cell division stages (t5, t5-t3, t5-t2, t8-t5) and blastocyst formation stages (tB-tSB) more quickly than aneuploid ones. If further studies confirm this, our results should serve as criteria for selecting embryos to be biopsied, potentially avoiding the procedure when euploidy can be predicted from morphokinetic features.

## INTRODUCTION

After more than 45 years since the first live birth resulting from in vitro fertilization (IVF) (Steptoe & Edwards, 1978), selecting the best embryo for transfer remains a challenge, particularly for women of advanced reproductive age, whose embryos are prone to aneuploidy. According to recent data on morphological embryo evaluation, blastocysts with an expansion grade ≥ 4 have a significantly higher chance of being euploid, as do those that combine grade A inner cell mass (ICM) and trophectoderm (TE) (Santamonkunrot *et al.*, 2024).

However, evaluation based on morphological criteria is static and overlooks timed and coordinated embryo development events that may provide insights into its health and implantation potential (Zaninovic *et al.*, 2017). The prospective cohort of Santamonkunrot *et al.* (2024) also showed that blastocysts with good cell division after genome activation-from day-3 to day-5-are likely to be euploid. In contrast, morphokinetic parameters obtained from sequential images, which can be generated by modern time-lapse (TL) incubators, are far from reliable predictors of live birth (Armstrong *et al.*, 2019).

According to a recent meta-analysis, TL imaging must still be considered an investigational procedure, with no established benefit on clinical outcomes when compared with conventional evaluation based on morphology (Jiang *et al.*, 2023). However, intriguing data support the need for continued investigation. In a prospective randomized controlled study, Goodman *et al.* (2016) suggested that the time to start blastulation, the absence of multinucleation, and the use of a score based on morphology and kinetics could be important predictors of blastocyst implantation. Additionally, it is known that the degree of fragmentation, multinucleation persisting to the four-cell stage, and the frequency of embryo contractions may be associated with a poorer reproductive prognosis, just as some morphokinetic variables may be significantly delayed in aneuploid embryos (Bamford *et al.*, 2022).

Morphokinetic parameters cannot be considered reliable markers of embryo ploidy; preimplantation genetic testing for aneuploidies (PGT-A) is the appropriate tool for selecting chromosomally normal embryos, which are supposed to be the best candidates for implantation leading to a healthy live birth. However, it is invasive and expensive, and the benefits may not be sufficient for young women (Cheng *et al*., 2022), especially since, in general practice, ploidy alone may not guarantee a good reproductive prognosis (Cimadomo *et al.*, 2023).

In situations where an individual marker is insufficient to identify the best embryo for transfer, the pursuit of a connection among morphokinetic variables, embryo developmental potential, and ploidy has increased in the literature. This rise is particularly tied to a growing interest in artificial intelligence (AI) tools, even though their widespread use for such predictions is not yet appropriate (Ma *et al.*, 2024). Recent data indicate predictive accuracy ranging from approximately 60% to 80% (Barnes *et al.*, 2023). Current evidence suggests that the combination of AI algorithms and PGT-A may be more beneficial for predicting favorable clinical outcomes than using either method in isolation (Lee *et al.*, 2024).

In our view, a cost-effective embryo selection tool that can non-invasively identify the reproductive competence of a blastocyst and prioritize only embryos with a predicted poor prognosis for PGT-A would signify a major breakthrough. Since such a tool does not yet exist, it seems premature to dismiss the potential of time-lapse imaging. This relatively new evaluation technique remains essential for studying morphokinetic parameters, aiming to discover a formula capable of shortening the time to pregnancy and protecting the best embryos from biopsies.

This study investigates, through the analysis of time-lapse images, whether specific morphokinetic parameters of cell division and blastocyst development reveal an embryo ploidy signature.

## MATERIALS AND METHODS

This retrospective observational study included 84 embryos undergoing preimplantation genetic testing for aneuploidies (PGT-A) from 18 couples attempting 25 intracytoplasmic sperm injection (ICSI) cycles between September 2022 and January 2024. The mean age of the female partners was 38.83±3.2 years, ranging from 22 to 44 years.

Time-lapse images from 41 euploid embryos (including four low-level mosaic embryos) and from 43 aneuploid embryos (including five high-level mosaic embryos) were analyzed for morphokinetic parameters based on cell division, as follows: the first evidence of cytoplasmic movements prior to the first cell division (st2); the time frame at which an embryo reaches a specified number of blastomeres (t2, t3, t4, t5, t8); the time frame at which an embryo initiates compaction (tSC); the time frame at which the blastocoel is first visible (tSB); the time frame at which the full blastocyst is formed (tB); and the intervals between some of them (cc3 [t5-t3], t5-t2, s3 [t8-t5], and tB-tSB).

Statistical analysis was conducted using GraphPad Prism 10, version 10.3.1 (GraphPad Software, LLC, Boston, MA, USA). Samples with a normal distribution were analyzed using Welch’s unpaired t-test, while samples with a non-parametric distribution were assessed with the Mann-Whitney test. Contingency analyses were carried out using Fisher’s exact test. Areas under the Receiver Operating Characteristic (ROC) curves were calculated for significant morphokinetic parameters to evaluate their ability to predict euploidy. The level of significance was set at *p*<0.05 for all analyses.

This protocol was approved by the Institutional Review Board of the Centro Universitário de Brasília - CEUB (Certificate of Presentation of Ethical Appreciation - CAAE n. 71313923.1.0000.0023, position statement n. 6.313.392), and all patients signed an informed consent form authorizing the use of their data.

## RESULTS

Among the TL parameters, t5, t2-st2, cc3 (t5-t3), t5-t2, s3 (t8-t5), and tB-tSB demonstrated a significant association with ploidy, being shorter for euploid blastocysts (Table 1).

**Table 1:** Timing of developmental events observed through time-lapse images in euploid and aneuploid blastocysts.

	Euploid (n=41)		Aneuploid (n=43)		95% CI	*p*
	n	Time (h)	n	Time (h)		
st2 (median)	33	22.6	38	22.3	-	0.8703
t2 (median)	41	24.7	43	25	-	0.4663
t3 (median)	41	36.6	43	36.2	-	0.5762
t4 (mean)	41	37.01	43	37.56	-0,9535 to 2,055	0.4685
t5 (median)	41	46.5	43	49.6	-	0.015
t8 (median)	41	53.8	42	54.55	-	0.4668
tSC (mean)	41	89.22	43	89.86	-3.771 to 5.062	0.5423
tSB (mean)	41	100.3	43	102.2	-2.341 to 6.046	0.3821
tB (mean)	41	108.3	43	112.2	-0.7682 to 8.557	0.2540
t2-st2 (median)	33	2.5	38	2.25	-	0.0236
cc2 (t3-t2) (median)	41	11.2	43	11	-	0.7367
cc3 (t5-t3) (median)	41	11.7	43	13.3	-	0.0046
t5-t2 (median)	41	22.1	43	24.4	-	0.0185
s2 (t4-t3) (median)	41	0.5	43	0.5	-	0.4058
s3 (t8-t5) (median)	41	10.9	42	3.3	-	0.019
tSC-t8 (mean)	41	34.59	42	33.42	-5.724 to 3.386	0.6106
tB-tSB (median)	41	7.6	43	9.9	-	0.0084

Legend: st2, first evidence of cytoplasmic movements prior to the first cell division; t2, t3, t4, t5, t8, time frames during which the embryo attains n number of blastomeres; tSC, time frame when the embryo begins compaction; tSB, time frame when the blastocoel becomes first visible; tB, time frame when the complete blastocyst is formed.

Additionally, cytoplasmic movements prior to the first cell cleavage were annotated; pattern 1 (vibration-like movements), pattern 2 (circular wave movements), and pattern 0 (the absence of detectable movements) showed no significant correlation with ploidy (Table 2).

**Table 2:** Cytoplasmic movement patterns before the first cell cleavage in euploid and aneuploid embryos.

	Euploid (n)	Aneuploid (n)	p
**Pattern 0**	8	5	0.4641
**Pattern 1**	17	23	
**Pattern 2**	16	15	

Patterns: 0, the absence of detectable movements; 1, vibration-like movements; 2, circular wave movements.

**Figure 1:**
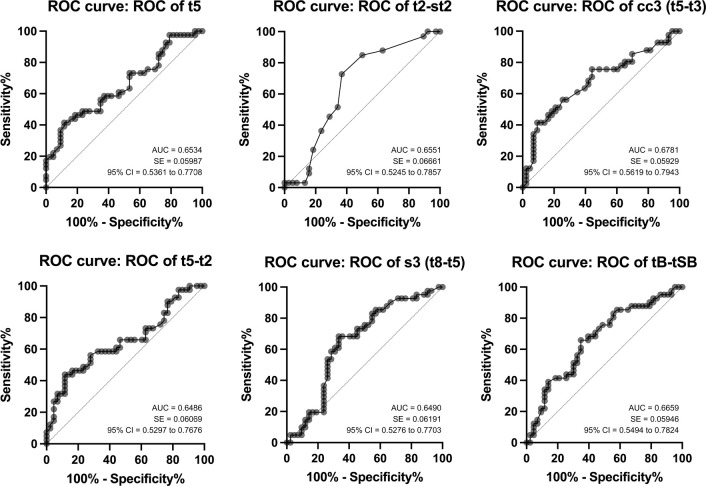
Receiver Operating Characteristic (ROC) curves for t5, t2-st2, cc3 (t5-t3), t5-t2, s3 (t8-t5), and tB-tSB predicting euploidy. AUC, area under the curve; SE, standard error; 95% CI, confidence interval.

A low predictive ability of those significant parameters to identify euploid embryos was found from the area under the ROC curves, with the interval cc3 and tB-tSB being the best ones (Figure 1).

## DISCUSSION

Despite the increasing number of research papers on their use in daily practice, the contribution of modern incubators and time-lapse (TL) images to the outcomes of assisted reproductive technologies remains uncertain. However, it is reasonable to suggest that evaluating embryo development based on the dynamics of cell division and blastocyst formation may provide more accurate information than static morphological evaluation. This study assessed the relationship between TL morphokinetic parameters and blastocyst ploidy; our findings indicated that faster cleavage and blastulation may be significantly associated with euploidy.

As a contribution to current knowledge, our study indicates that the time intervals an embryo takes from three to five blastomeres, and from the first identification of blastocoel to full blastocyst, appear to be the main TL variables to consider in predicting ploidy. In fact, such findings can be implemented in clinical practice, enhancing embryo selection as a cost-effective tool that integrates TL, especially for patients with limited resources-a common condition in Brazil-and/or as an informative method to select embryos that could be spared from biopsy. It is known that, to date, science lacks information on how morphokinetic parameters could be used alongside traditional grading systems to improve embryo selection. We consider our findings a catalyst for further investigation into the use of TL in routine practices. Additionally, our results align with recent data.

In the study by Serrano-Novillo *et al.* (2023), in addition to the synchronicity and sequentiality of cleavages observed in euploid embryos, they developed significantly faster than aneuploid ones, which exhibited longer st2 (the first evidence of cytoplasmic movements prior to first cytokinesis) (1.5±0.9 *vs*. 1.6±0.7), t3 (12.9±3.4 *vs*. 13.6±2.6), t5 (25.5±6.1 *vs*. 27.1±4.5), tSB (73.8±7.0 *vs*. 76.3±7.5), tB (83.6.5±7.4 *vs*. 86.2±7.6), cc3 (t5-t3) (12.5±4.8 *vs*. 13.6±2.9), and t5-t2 (22.9±6.2 *vs*. 24.4±4.7).

Minasi *et al.* (2016) analyzed 1730 biopsied blastocysts. They found that cleavage from the threeto four-cell stages (s2), the time to the four-cell stage (t4), the time to blastocyst formation (tSB), the time to reach the full blastocyst stage (tB), the time to expand (tEB), and the time to hatch (tHB) were significantly faster in euploid embryos compared to aneuploid embryos: s2, 2.6 hours *vs*. 4.2 hours; t4, 40.0 hours *vs*. 41.1 hours; tSB, 103.4 hours *vs*. 105.0 hours; tB, 110.2 hours *vs*. 112.8 hours; tEB, 118.7 hours *vs*. 122.1 hours; and tHB, 133.2 hours *vs*. 137.4 hours, respectively.

A delayed start time of blastulation (tSB ≥ 96.2 hours), expansion (tEB > 116 hours), and tEB-tSB intervals longer than 13 hours were markers for aneuploidy. However, the finding of faster development in euploid embryos is not unanimous. Patel *et al*. (2016) retrospectively compared euploid and aneuploid embryos and found similar blastulation rates as well as morphokinetic behaviors between them. Similarly, in a retrospective analysis of 256 blastocysts, no significant differences were found in developmental rates, except for a longer blastocyst expansion interval (tEB-tB) in aneuploid embryos, which was insufficient to improve the chance of selecting a chromosomally normal embryo for transfer (Zhang *et al.*, 2017).

It must be clear that static morphology classification still matters. Initial studies on embryo morphokinetic behavior in time-lapse found that high-quality inner cell mass (ICM) was more frequent among euploid blastocysts compared to aneuploid ones (Minasi *et al.*, 2016), as well as the combination of two or more dysmorphisms, which was frequently associated with aneuploidy (Desai *et al.*, 2018). In the prospective analysis of low-quality blastocysts according to expansion, inner cell mass, and trophectoderm (Gardner’s criteria), ploidy could not be correlated to morphokinetic parameters; regardless of being euploid or aneuploid, low-quality blastocysts intriguingly cleaved up to 5 cells faster than high-quality ones (48.4 *versus* 50.2 hours, respectively) and progressed slowly in other developmental markers, such as morula (91.5 *versus* 88.3 hours, respectively) and blastocyst formation (114.0 *versus* 106.9 hours, respectively) (Quinn *et al.*, 2022). This aligns with the previously mentioned study of Santamonkunrot *et al.* (2024), which also demonstrated that grade B TE may be significantly associated with a higher chance of euploidy than grade C.

Before the advent of artificial intelligence (AI), morphokinetic parameters were limited to being part of the prognostic puzzle, used alongside genetic assessment for blastocyst selection (Minasi *et al.*, 2016; Desai *et al.*, 2018). The early-reported limitations of time-lapse technology decision-making-operator dependence and the necessity to incorporate clinical and preimplantation genetic testing information-may have been, at least in part, resolved by the introduction of AI into the interface between clinical and laboratory practices.

One limitation of our study may be the lack of differentiation between euploid and low-level mosaic blastocysts, as well as aneuploid and high-level mosaic blastocysts. Although mosaic embryos may display morphokinetic parameters that overlap with those observed for euploid and aneuploid embryos (Martín *et al.*, 2021), the literature is controversial and unclear regarding differences in the developmental pace of lowand high-level mosaic embryos (Zou *et al.*, 2024). Additionally, in our population, the analysis conducted after excluding mosaic embryos yielded similar numbers, prompting us to include these embryos according to the current trend of considering low-grade mosaic embryos as suitable for transfer, while high-grade ones are deemed unsuitable (Capalbo *et al.*, 2021).

Other limitations of our study include its retrospective design and small population size, which limit the precision and reliability of our conclusions, leading readers to interpret non-significant findings cautiously. However, these limitations do not undermine the role of our results in composing a scenario where certainties are scarce and new insights are welcome. Finally, it is worth noting that we are currently conducting a prospective study to validate the presented findings and obtain more precise effect estimates.

In conclusion, euploid blastocysts appear to develop faster than aneuploid ones, according to TL morphokinetic parameters/intervals t5, t2-st2, cc3 (t5-t3), t5-t2, s3 (t8-t5), and tB-tSB, although they show low predictive ability in our population. While PGT-A is currently the most accurate tool for assessing embryo ploidy, the predictive potential of these morphokinetic parameters suggests that they should be incorporated into conventional grading systems and artificial intelligence algorithms, potentially enhancing embryo selection. Finally, these morphokinetic parameters/intervals should ultimately be considered as criteria for selecting embryos for biopsy, possibly allowing us to forgo the procedure when euploidy is predictable. However, further larger prospective studies or subgroup analyses of mosaic embryos in a larger cohort, even retrospectively, are necessary to validate our findings.
